# The Sound Environment in the PICU: A Scoping Review Describing Sound Levels, Interventions and Patient Impacts

**DOI:** 10.1111/jpc.70591

**Published:** 2026-09-11

**Authors:** Janeen Bower, Hazel Low, Naomi Khan, Sebastian J. Corlette, Emma Macdonald‐Laurs, Andrea Veysey, Ben Gelbart, Kate Masterson

**Affiliations:** ^1^ Faculty of Fine Arts and Music The University of Melbourne Melbourne Victoria Australia; ^2^ Department of Music Therapy The Royal Children's Hospital Melbourne Melbourne Victoria Australia; ^3^ Clinical Sciences Murdoch Children's Research Institute Melbourne Victoria Australia; ^4^ Department of Clinical Technology The Royal Children's Hospital Melbourne Melbourne Victoria Australia; ^5^ Paediatric Intensive Care Unit The Royal Children's Hospital Melbourne Melbourne Victoria Australia; ^6^ Department of Paediatrics The University of Melbourne Melbourne Victoria Australia; ^7^ Department of Anaesthesia and Pain Management The Royal Children's Hospital Melbourne Melbourne Victoria Australia; ^8^ Department of Critical Care The University of Melbourne Melbourne Victoria Australia; ^9^ Department of Neurology The Royal Children's Hospital Melbourne Melbourne Victoria Australia

**Keywords:** auditory stimulation, critical care, hospitalised child, noise, sound

## Abstract

Elevated sound levels are associated with increased morbidity in adult and neonatal intensive care; however, their impact in the paediatric intensive care unit (PICU) remains poorly characterised. The aim of this scoping review was to characterise sound exposure in the PICU, including its measurement, sources, and associations with patient outcomes. MEDLINE, Embase, PsycINFO (Ovid), and PubMed were searched up to 5 November 2025 for full‐text, peer‐reviewed publications within the past 10 years to reflect the contemporary PICU environment. Titles, abstracts, and full texts were independently screened by two authors, and results were reported according to the PRISMA Extension for Scoping Reviews. Thirty‐six publications were included. Publications were grouped into five domains: measurement of sound levels; sources of sound; patient impacts; sound reduction interventions including care bundles incorporating sound descriptions; and other. Reported sound levels varied substantially across studies, reflecting heterogeneity in measurement approaches and clinical contexts. Most studies reported sound levels above WHO recommendations, and several reported maximum levels exceeding 80 dBA. Sound sources were broadly categorised as medical equipment, human activity, and environmental factors. Links between elevated sound and clinically meaningful outcomes remain uncertain. Intervention studies were limited, often behaviour‐focused, and rarely evaluated patient‐centred outcomes or clinical sustainability. Current evidence suggests WHO recommended sound levels are rarely achieved. The sound environment in the PICU is multifaceted, reflecting the interaction of clinical equipment, human activity, and environmental systems. There is a need for interventional research to evaluate whether modification of the PICU sound environment meaningfully improves patient outcomes.

## Key Points

- The PICU sound environment is consistently above WHO recommended levels and reflects a combination of equipment, human activity and environmental factors, requiring a broader approach than simple noise reduction strategies.
- Current evidence provided limited links between sound exposure and clinically meaningful outcomes, limiting the ability to implement targeted, evidence‐based interventions in routine care.
- Future approaches should move beyond noise reduction to differentiate harmful from meaningful sound, supporting strategies that minimise environmental stressors and optimise beneficial auditory inputs.

## 1 Introduction

The paediatric intensive care unit (PICU) is a complex, high resource environment. Mechanical ventilators, alarms, medical and cleaning equipment, staff, families and patients generate a potentially harmful sound environment [1]. Sound pressure level is a logarithmic measure of the ratio of a given sound pressure to a reference, expressed in decibels (dB) [2]. Sound is often reported using the A‐weighted measurement (dBA) which filters frequency to approximate human hearing. As reporting is inconsistent across PICU publications, use of dB or dBA in this manuscript follows the original source. The term *noise* introduces subjectivity, referring to unpleasant sound experiences shaped by social and cultural factors [2]. The perception of sound is also influenced by additional factors including frequency and individual temporal processing [3].

The World Health Organisation (WHO) recommends that *L*_Aeq_ (average A‐weighted sound level over a specified period of time) background sound in hospital rooms not exceed 35 dB [4] and the Association of Australasian Acoustical Consultants recommends that the *L*_Aeq_ PICU not exceed 45 dB [5]. Noise‐related sleep disruption is well documented in adult ICU, resulting in slower recovery and prolonged hospitalisation [6]. Further, evidence suggests that excessive nighttime sound exposure may increase delirium risk in hospitalised children [7, 8]. The impact of sound exposure has been extensively examined in the NICU because of concerns about disrupted cortical maturation in preterm infants [9]. In the wider healthcare setting, noise is associated with a reduction in sleep which can impact recovery and a cumulative exposure of excessive sound can impact children later in life [10, 11]. However, research in the PICU remains fragmented, highlighting the need for this review.

A scoping review methodology was selected to consolidate available evidence, highlight knowledge gaps, and inform future research. The flexible nature of this approach supported an iterative process of data extraction and analysis [12, 13]. The review aimed to:

1. Describe current PICU sound levels
2. Describe sound modification interventions
3. Describe the effects of sound exposure on patients
4. Describe major contributors to sound levels

The population of interest was paediatric patients, the concept was sound exposure and sound mitigation, and the context was the paediatric intensive care unit.

## 2 Methods

### 2.1 Context

The authors represent a multidisciplinary team across music therapy, nursing, PICU, anaesthesiology, neurology and clinical technology. This implicitly shaped data analysis and interpretation.

### 2.2 Data Sources

The scoping review was conducted using the JBI methodology for scoping reviews, reported according to the PRISMA Extension for Scoping Reviews [13, 14, 15], and registered with OSF (https://doi.org/10.17605/OSF.IO/BGTE2). Database search strategies were developed with a medical research librarian and included MEDLINE (Ovid), Embase (Ovid), PsycINFO (Ovid). PubMed was also searched to capture publications not indexed in Medline. The Medline and PsycINFO search strategies are included in Appendices A and B. Additional publications were identified through backward and forward citation tracking. References were exported to Covidence (Veritas Health Innovation, Australia) for screening.

### 2.3 Inclusion Criteria

We included English language (or translated), full‐text publications from 2015 to 2025 conducted in PICUs to reflect the contemporary sound environment. We excluded publications based in neonatal settings, involving infants < 36 weeks postmenstrual age (PMA) or in adult ICU populations (> 18 years). Where publications included a mixed sample (e.g., NICU and PICU), we extracted data only for the PICU cohort.

### 2.4 Source Screening and Data Synthesis

All authors contributed to the screening process. Titles and abstracts were independently reviewed by two reviewers, with discrepancies resolved by a third. Full‐text articles were reviewed by two reviewers, with consensus reached through team discussion. Data extraction was undertaken in two stages [13, 14]. First, general publication characteristics were extracted into an Excel template. Additional categories were inductively identified from the data. All data were checked by a minimum of two authors. Data were descriptively analysed. Finally, findings were interpreted in relation to the reviewers' clinical and research expertise to inform the review.

## 3 Results

Database searches were conducted on 4 November 2024 and returned 4118 records. After removing duplicates, 2715 titles and abstracts were screened, and 64 full‐text articles assessed. Thirty‐one publications were included. An additional publication was identified through reference list and citation screening. Updated searches were conducted on 5 November 2025 and identified four further publications (see Figure 1). The 36 included publications are detailed in Table 1.

**FIGURE 1:**
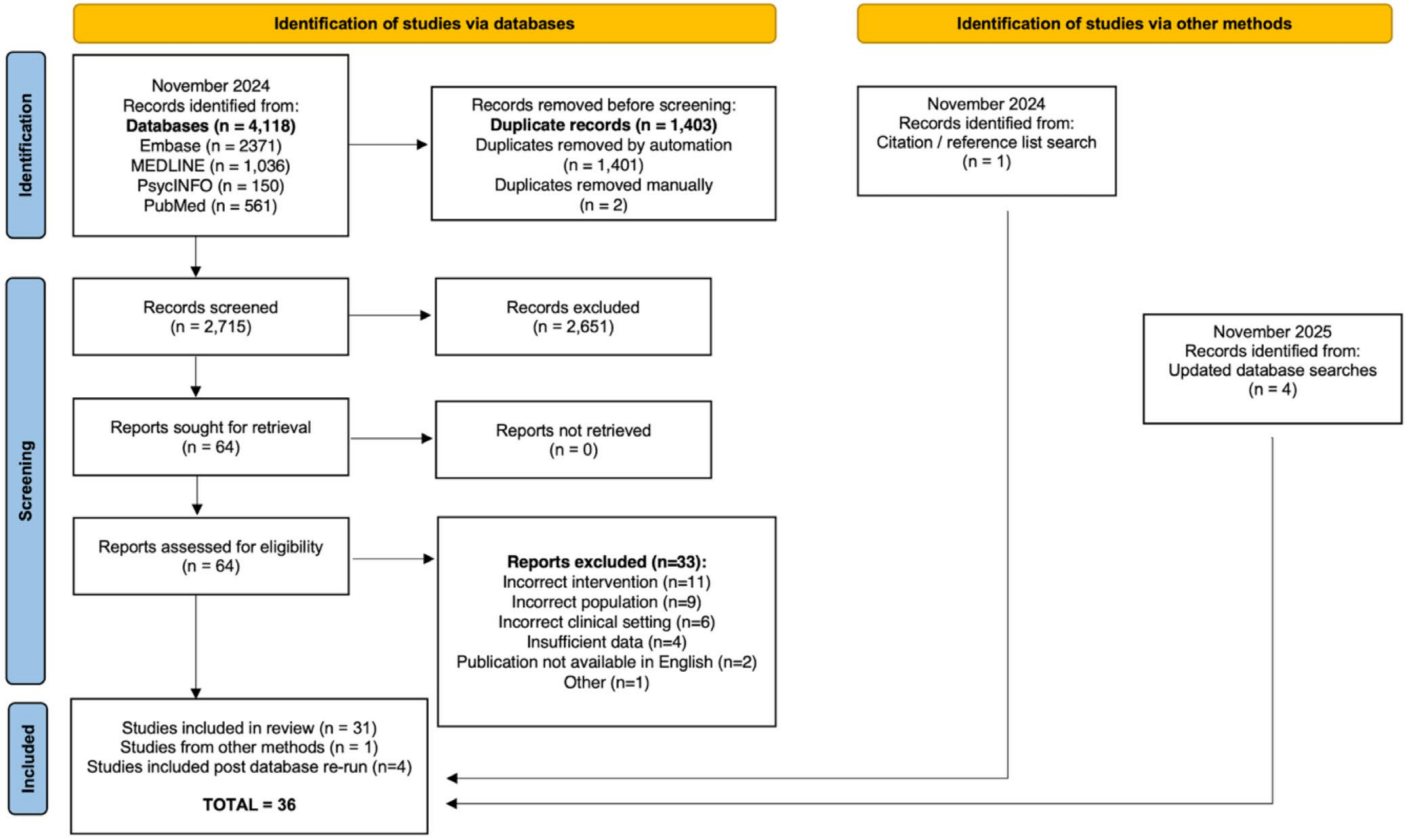
PRISMA diagram of publication selection.

**TABLE 1:** Summary of included publications.

Author	Year	Study focus	Location	Lead author	Methodology	Participants	Mechanical ventilation
Abuqamar, et al.	2016	Other	Jordan	Nurse	Survey	*n* = 123 Parents only	NA
Bosch‐Alcaraz, et al.	2021	Sound measurement	Spain	Nurse	Observational	NA	Yes
Butler, et al.	2024	Care bundle	United States (US)	Psychologist	Quality project	*n* = NR Age: 0–3 months	Yes
Caprarola, et al.	2024	Sound measurement Patient impacts	US	Doctor of Medicine (MD)	Observational	*n* = 68 Age: 0–18 years	NR
Cardoso, et al.	2021	Other	Brazil	Not reported	Theoretical	NA	NA
Curley, et al.	2024	Care bundle Sound measurement	US	Nurse	Cohort	*n* = 54 Age: 6 months – 17 years	Yes
Daniels and Harrison	2016	Sound measurement	US	Nurse	Observational	*n* = 1 Age: 10 days	No
Disher, et al.	2017	Sound measurement Patient impacts Intervention	Canada	Nurse	Mixed methods	*n* = 15 Staff *n* = 2 Parents	NR
Garcia Guerra, et al.	2018	Sound measurement Patient impacts	Canada	MD	Observational	*n* = 39 Age: 5.5 months (median)	Yes
Garcia Guerra, et al.	2016	Other	Canada	MD	Survey	*n* = 98 Intensivists	NA
Garrido Galindo, et al.	2016	Sound measurement	Colombia	Nurse	Observational	*n* = not reported (NR) Age: 31 days—14 years	NR
Greenfield, et al.	2020	Sound measurement	US	MD	Observational	*n* = 100 Age: 0–18 years	Yes
Greenfield, et al.	2023	Patient impacts	US	MD	Observational	*n* = 142 Age: 1.6 years (median)	Yes
Hassinger, et al.	2024	Patient impacts Sources of sound	US	MD	Survey	*n* = 152 Parents	NR
Jung, et al.	2020	Sound measurement Sources of sound	Korea	MD	Observational	NA	Yes
Kalvas and Harrison	2024	Sources of sound	US	Nurse	Observational	*n* = 12 Age: 1–4 years	No
Kalvas and Harrison	2020	Other	US	Nurse	Feasibility	*n* = 3 Age: < 30 days	No
Kalvas, et al.	2023	Sound measurement Patient impacts	US	Nurse	Observational	*n* = 10 Age: 1–4 years	No
Kalvas, et al.	2022	Other	US	Nurse	Feasibility: Protocol	*n* = 10 Age: 1–4 years	No
Kaur, et al.	2016	Sound measurement Sources of sound Intervention	US	MD	Survey	*n* = 50 Families	Yes
Kawai, et al.	2019	Care bundle Sound measurement	US	MD	Quasi‐experimental	*n* = 8 Age: 2.1 years (median)	Yes
Kol, et al.	2015	Sound measurement Sources of sound Intervention	Turkey	Nurse	Quasi‐experimental	NA	Yes
Konca, et al.	2022	Patient impacts	Turkey	MD	Survey	*n* = 19 Physicians	NA
Kramer, et al.	2016	Sound measurement Sources of sound	US	MD	Observational	*n* = 100 Age: 0–26 years	Yes
Kudchadkar, et al.	2016	Sources of sound	US	MD	Survey	*n* = 90 Nurses	NA
Kudchadkar, et al.	2014	Patient impacts	US	MD	Survey	*n* = 341 Intensivists	NA
Nenningsland, et al.	2024	Patient impacts Sources of sound	Norway	Nurse	Survey	*n* = 10 Parents	NR
Nenningsland, et al.	2025	Other Sources of sound	Norway	Nurse	Observational Cross‐sectional	*n* = 79 Nurses	Yes
Nenningsland, et al.	2025	Sound measurement Patient impacts Sources of sound	Norway	Nurse	Observational Cross‐sectional	*n* = 21 Age: 5 months—5 years	Yes
Shoemark, et al.	2016	Sound measurement	Australia	Music Therapist	Observational	NA	Yes
Sood, et al.	2016	Other	US	Psychologist	Survey	*n* = 28 Staff	NR
Stremler, et al.	2021	Patient impacts	Canada	Nurse	Cross‐sectional	*n* = 69 Age: 1–18 years	NR
Waterson and Bedner	2019	Sources of sound	Switzerland	Scientist/Engineer	Retrospective data mining	NA	NR
Watson, et al.	2015	Sound measurement Sources of sound Other	US	Scientist	Observational	*n* = 15 Nurses	NR
Weatherhead, et al.	2022	Sound measurement Patient impacts	US	MD	Observational	*n* = 1114 Age: 12.1 years (median)	Yes
Witte, et al.	2023	Patient impacts	US	MD	Survey	*n* = 30 Age: 6.5 years (median)	Yes

Lead authors were predominantly medical doctors and registered nurses, and approximately half of the included publications originated from the United States. Twenty (of 36) publications explicitly reported on ventilation status, and 15 of these included mechanically ventilated patients. Twenty‐eight publications described general PICUs, six described paediatric cardiac ICUs (CICU), and two included data from both PICU and CICU/NICU. Most publications involved observational (16/36) or survey (10/36) study designs.

Five domains of focus emerged through the iterative process of data extraction and synthesis. The publications were grouped according to their primary foci: measurement of sound levels; sources of sound; patient effects; noise‐reduction interventions plus care bundles incorporating descriptions of sound; and other (see Figure 2). Publications could contribute to multiple domains.

**FIGURE 2:**
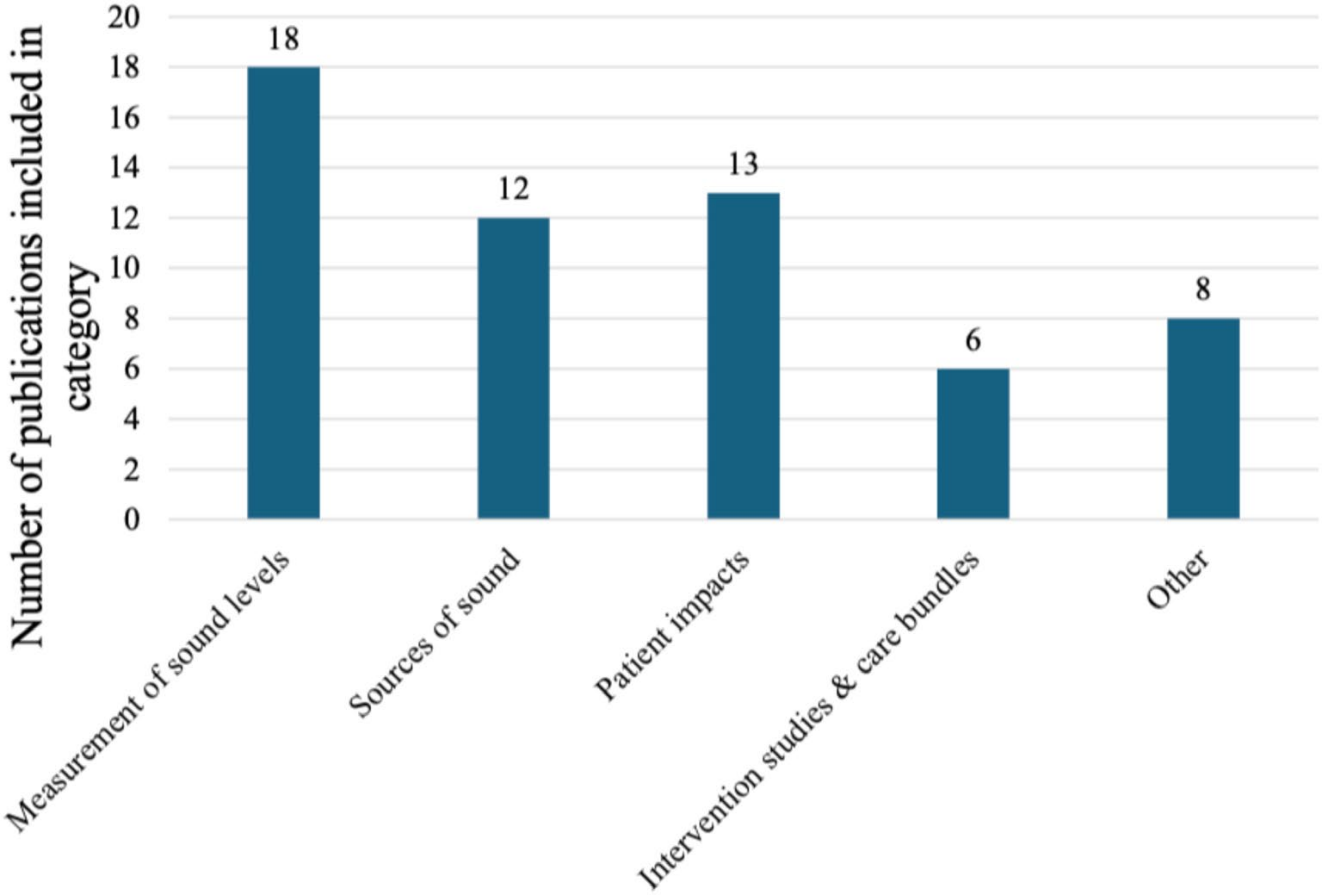
Grouping of publications into domains of focus.

### 3.1 Measurement of Sound Levels—18 Publications [1, 8, 16, 17, 18, 19, 20, 21, 22, 23, 24, 25, 26, 27, 28, 29, 30, 31]

Cross‐study comparability of data was limited by heterogeneity across clinical context, sampling periods, measurement devices and positioning of the measurement devices. Six publications reported sound measurement in dB and the remaining 12 reported dBA. Conversion of these data for comparison is not possible without knowing the underlying frequency spectrum of the sound. Summary statistics for central tendency of data were variably reported using median or mean. The median sound level describes what the patient typically experiences, whereas the mean reflects the total cumulative sound burden over time by incorporating intermittent peaks.

Only one publication reported a measurement below WHO recommendations. Nenningsland et al. [29] recorded a minimum 33 dBA in a single room yet median noise levels exceeded 35 dBA for 99.87% of the time. Kawai et al. recorded the lowest mean sound level (44 dB), captured in an empty patient room [26]. Daniels and Harrison [19] reported levels that appear inconsistent with the clinical context of an awake post‐surgical infant in a 10‐bed CICU, raising concerns about instrument sensitivity, calibration, or data analysis errors, limiting interpretability of findings.

Extremely high sound levels were also recorded. Watson et al. [31] reported a mean of 73.8 dBA from on‐body recordings from nurses. Maximum sound levels reached 101 dBA at the nurses' station in Disher et al. [1], 95 dBA in single and shared rooms in Garrido Galindo et al. [21] and 134.5 dBA in Kramer et al. [28], which is approaching the threshold for physical pain. Kramer et al. [28] observed peak levels above 100 dBA for an average of 115 min per day.

Heterogeneity in frequency weighting means that data cannot be meaningfully compared between studies reporting dB and those reporting dBA. Additionally, studies variably reported data for different conditions. Accordingly, a descriptive approach using box‐and‐whisker plots was used for visual comparison of the range and variability of reported sound levels within each unit category (see Figures 3 and 4). These are presented separately for dB and dBA to avoid inappropriate quantitative comparison. Daniels and Harrison [19] were excluded due to concerns regarding measurement veracity.

**FIGURE 3:**
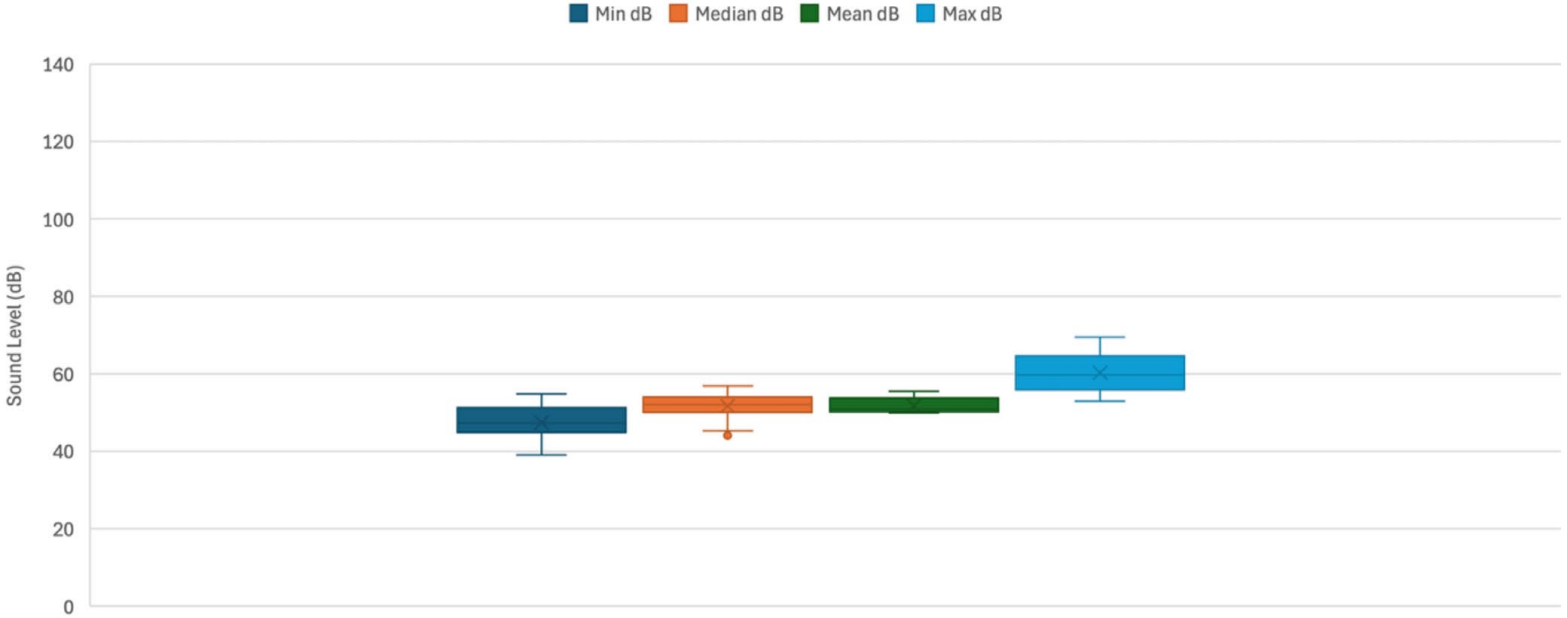
Distribution of unweighted decibel measurements (dB) across included studies (6 publications).

**FIGURE 4:**
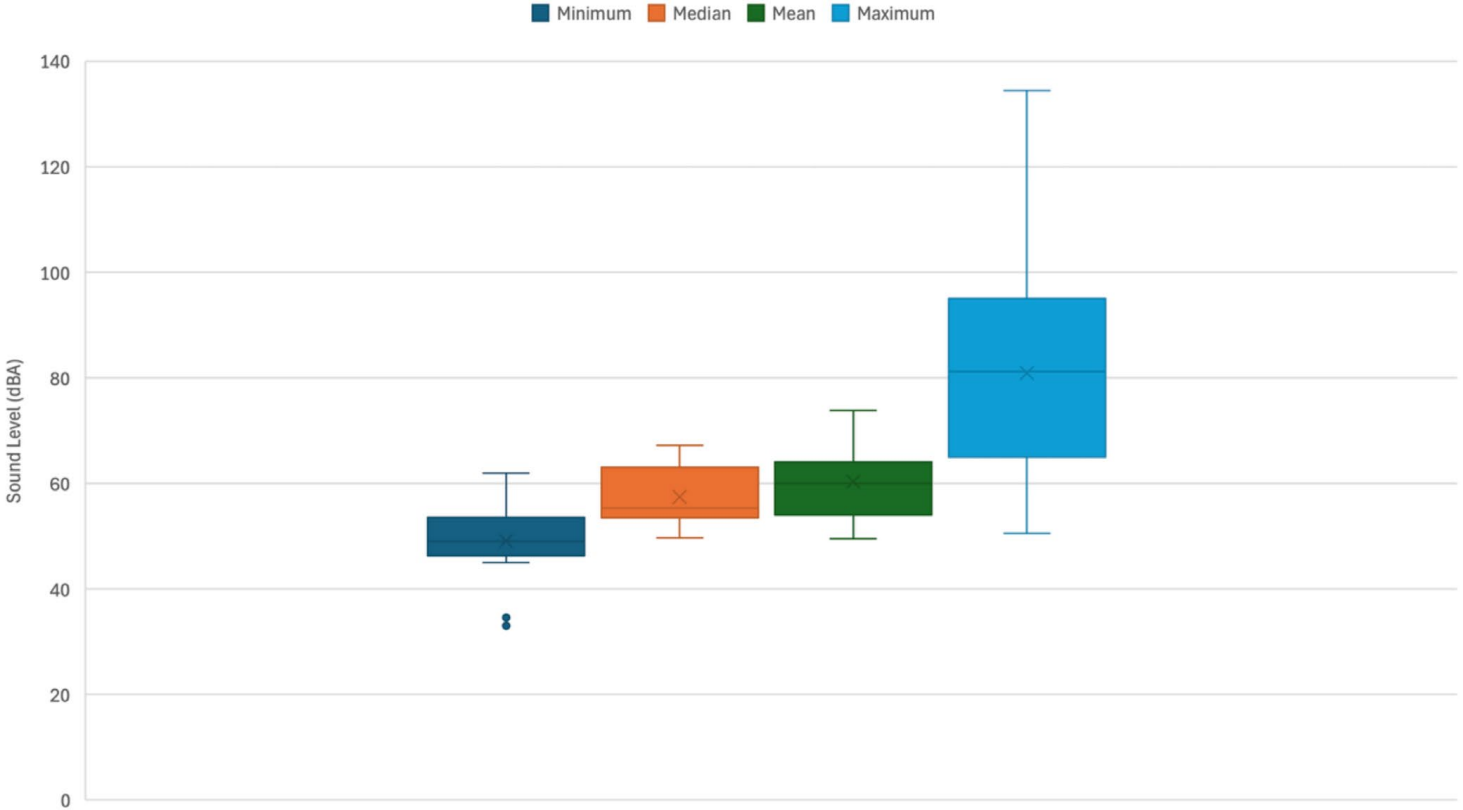
Distribution of A‐weighted decibel measurements (dBA) across included studies (11 publications).

The mean of study minimums was 47 dB for studies reporting in dB and 49 dBA for studies reporting in dBA. Median values reflect the sound levels typically experienced by patients, whereas mean values reflect cumulative sound burden over time. The mean of study medians was 52 dB for studies reporting in dB and 57 dBA for studies reporting in dBA. The mean of study means was 52 dB and 60 dBA, respectively. For studies reporting in dBA, this higher mean relative to the median may reflect the influence of repeated intermittent peak events. Peak exposures themselves vary widely, with more than half of publications that reported dBA measures documenting peak levels exceeding 80 dBA. Mean peak levels were 60 dB and 81 dBA for the dB and dBA categories, respectively.

### 3.2 Sources of Sound—12 Publications [23, 25, 27, 28, 29, 31, 32, 33, 34, 35, 36, 37]

Sound sources were grouped into medical equipment, human activity and environmental sources.

*Medical equipment* was the primary contributor to loud and frequent sound events. Objective measurements identified equipment alarms (e.g., ventilator and monitor) exceeding 70–80 dBA [23, 27], with wall suction under obstruction up to 91 dBA [23]. Nebulisers, oxygen delivery systems and warming devices generated approximately 50–70 dBA [23, 27, 31]. Perception‐based studies reflected similar patterns. Parents and nurses identified alarms and bedside machinery as major contributors to sound [28, 29, 32, 33, 35, 36]. Waterson and Bedner [37] demonstrated a higher infusion pump alarm burden in the PICU compared with other hospital units and raised concerns about alarm fatigue and potential impacts on staff vigilance.

*Human activity* also accounted for a substantial proportion of sound. Sound at the nurses' station reached 91 dBA [27], with staff and family communications recorded around 70 dBA [23, 27, 31]. Nurses identified staff communication as an intrusive noise source [34], and parents reported distressed patients and clinical communication as contributors [28, 29, 32, 35]. Kalvas and Harrison noted that not all human‐generated sound was perceived negatively, with a consideration that family voices may act as a buffer for stress [33].

*Environmental sounds* including doors, hallway traffic, telephones and maintenance activities contributed to the persistent background sound measuring 50–70 dBA [25, 27, 31]. Media‐related noise was also prominent and included televisions, radios, and tablets/iPads [33]. Although less intense than equipment, environmental sounds were unpredictable.

### 3.3 Patient Effects of Sound Exposure—13 Publications [1, 8, 17, 20, 24, 29, 32, 35, 38, 39, 40, 41, 42]

*Delirium*: Four studies examined associations between sound exposure and delirium using the Cornell Assessment of Paediatric Delirium (CAPD). Caprarola et al. [17], Greenfield et al. [38] and Kalvas et al. [24] found no significant association, while Weatherhead et al. [8] identified a significant positive correlation between nighttime sound levels and CAPD scores (*r* = 0.6; *p* < 0.001). Konca et al. [42] reported that 21% of survey respondents implemented noise‐reduction strategies using sound‐measuring devices as a non‐pharmacological approach to delirium management.

*Sedation use*: Two publications reported significant associations between sound exposure and sedation requirements. Higher average and peak nighttime sound levels were associated with increased sedative use, with each 1 dB rise associated with a 1.07‐fold increase in likelihood of sedation, which included benzodiazepines, opioids, and dexmedetomidine (*p* < 0.01) [17]. Similarly, elevated sound levels were associated with greater PRN sedation needs within 2–5 h (*p* = 0.03) [20].

*Physiological variables*: Elevated average and peak sound levels were associated with increases in heart rate and blood pressure (*p* < 0.001) [17]. Higher noise levels were also associated with oxygen desaturation events [1].

*Sleep*: Kalvas et al. reported no clear association between sound exposure and sleep efficiency measured using video monitoring and actigraphy [24]. In contrast, Stremler et al. reported that sound events exceeding 80 dB increased the risk of “awakening” (*p* = 0.04) [40]. Similarly, Nenningsland et al. reported noise caused 11% of awakenings [29]. Subjective reports varied with caregivers perceiving that louder nighttime environments compromised sleep (*p* = 0.02), whereas nursing staff did not report a similar effect [41]. In parent‐led surveys, environmental noise was identified as a contributor to sleep disturbance [32].

### 3.4 Intervention Studies Plus Care Bundles—Six Publications [1, 18, 25, 26, 27, 43]

Kol et al. [27] reported lower daytime sound levels in single rooms in a newly remodelled PICU compared with the four‐bed unit in the original PICU (56.0 dBA vs. 72.1 dBA; *p* < 0.05), with evening and nighttime sound levels also 10 dBA and 7 dBA lower, respectively. Kaur et al. and Disher et al. identified closing doors as the most effective sound‐reduction intervention [1, 25], and suggested rationalising the number of alarms and alarm volume as additional strategies. Visual reminders, including signage, were also described as effective [1, 25].

The remaining three publications in this domain evaluated sound‐reduction strategies as part of broader care bundles. Care bundles are structured sets of interventions that ensure consistent delivery of best practice [44]. Sound interventions within care bundles focused on reducing noise, moving staff interactions away from bedside and closing doors. Two bundles [18, 43] also incorporated meaningful sounds by encouraging staff and families to speak softly and calmly. Implementation of care bundles resulted in reduced perceived and/or measured sound levels compared to baseline [43] or comparison groups [18, 26]. Curley et al. also reported improved circadian rhythm alignment prior to extubation [18].

### 3.5 Other—Eight Publications [31, 36, 45, 46, 47, 48, 49, 50]

Kalvas et al. [50] reported their sound study protocol was time and resource intensive, with < 50% recruitment, and Kalvas and Harrison [48] noted mixed feasibility of continuous environmental data capture. Watson et al. [31] found nurses' heart rate positively correlated with sound levels, although nurses did not report increased stress. A further theoretical paper drew on Florence Nightingale's Environmental Theory highlighting that sound has historically been recognised as influencing recovery [46].

One publication reported that 60% of surveyed Canadian intensivists (*n* = 98) did not use noise reduction strategies [47]. In contrast, another found that 21% of PICUs implemented designated quiet times [49]. A third survey reported that > 50% surveyed parents (*n* = 123) were dissatisfied with PICU noise levels and felt it impacted their child's rest [36]. A final survey indicated that nurses perceived multibed units as inferior to single rooms for controlling noise [45].

## 4 Discussion

In this scoping review of 36 publications, median sound levels in the PICU were approximately 52 dB (studies reporting dB) and 57 dBA (studies reporting dBA), and several peaks exceeded 80 dBA. Interventions to modify the sound environment were limited and included alarm management and environmental modifications, with few approaches addressing the optimisation of meaningful sound. Patient impacts associated with sound exposure included increased sedation requirements, cardiovascular responses, oxygen desaturation, and sleep disruption. Collectively, these findings indicate that while there is widespread concern that typical sound environments in the PICU may be harmful, their measurement, modification, and clinical implications remain incompletely characterised. However, as this was a scoping review, the findings should be interpreted as a map of the available evidence rather than an assessment of intervention effectiveness or causal relationships.

### 4.1 The PICU Sound Environment Is Loud

The sound environment is multifaceted, arising from clinical equipment, human activity, and background environmental sounds. Building systems, patient ventilation, and equipment provide a continuous baseline sound, captured in empty patient rooms, for example by Kawai et al. [26]. The WHO recommendation of an *L*_Aeq_ of 35 dB was not achieved during routine care. The typical sound experience, reflected in median sound levels, sits well above the WHO recommendation [23, 28, 29]. Peak exposures were reported to be high, with several publications reporting peak levels exceeding 80 dBA in studies that reported dBA [24, 28, 29]. Sound levels were generally lower at night but remained above threshold recommendations [17, 18, 20]. Sound peaks occurred across day and night and likely contribute to physiological arousal, highlighting potential clinical relevance.

Sound level differences observed between empty and occupied rooms, and between day and night, suggest that it may be possible to distinguish modifiable from non‐modifiable sound sources. Human activity and workflow may be amenable to modification of communication practices, equipment handling and alarm management, offering opportunities to reduce overall sound levels. Only three publications evaluated strategies to reduce PICU sound levels, and the only publication that reported pre and post‐intervention sound measurements involved substantial building redesign [27]. Other proposed strategies, including staff education, visual reminders, alarm management, and designated quiet times, have been described, but their effect on sound levels has not been quantified [1, 25]. Evidence from NICU settings suggests that educational strategies and behavioural prompts may contribute to modest reductions in sound levels, although the environments frequently remain above recommended thresholds [51].

### 4.2 Patient Effects of Sound Exposure Are Incompletely Characterised

High sound levels are increasingly recognised as potential environmental stressors in the adult ICU [52], yet the association in paediatric critical care is incompletely characterised. Sleep disturbance was the most frequently reported patient variable, with disruption of sleep continuity and reduced sleep duration associated with increased disease burden, longer length of stay, and long‐term sequelae in paediatric critical illness [53]. Of the seven publications that examined the associations of sound and sleep, three utilised actigraphy [24, 29, 40] and four relied on parent or nursing report [32, 35, 39, 41]. However, ventilation and sedation status were inconsistently reported, and observational assessments of sleep may be unreliable in sedated or mechanically ventilated patients. Therefore, any association between sound exposure and sleep in these critically ill children is especially unclear.

A few small studies reported associations between sound exposure and delirium, sedation requirements, or altered physiological parameters. However, most studies did not adequately account for important confounders, including illness severity, medications and timing of admission, limiting interpretation of causal relationships between sound exposure and patient outcomes. One publication reported a positive association between higher nighttime sound levels and delirium risk [8], while two small studies reported an association between increased sound levels and a greater probability of sedation use (*n* = 68; *n* = 39) [17, 20]. However, the paucity of these findings reflects the complexity of the PICU environment, where delirium and stress are associated with many factors including illness severity, medications, and sleep disruption. This makes it difficult to isolate the contribution of sound.

The medium‐ and long‐term effects of exposure to loud and potentially noxious sound environments in the PICU remain largely unexplored. No studies examined associations with length of stay, duration of mechanical ventilation, psychological impacts of PICU admission, post‐ICU distress, family wellbeing during admission, the incidence of agitation or aggression, or longer‐term neuropsychological sequelae including cortical maturation.

### 4.3 Not All Sound Is Noise

A recurring theme in this review was the assumption that all elevated sound in the PICU is noise. Only Kalvas and Harrison [33] acknowledged that sounds including family voices may be positive sounds. Similarly, care bundles described the use of familiar voices or white noise to mask sudden sound peaks [18, 26, 43]. A more nuanced understanding of the PICU sound environment is needed.

Individual perception of sound considers more than just the dB level of the sound; for example, a sound of the same dB but of different frequency will not be perceived the same [3]. This distinction is particularly relevant in the PICU, where monitor alarms, ventilators, conversations, and equipment each have distinct acoustic profiles despite often occurring within similar overall sound pressure ranges. Furthermore, structured, meaningful sounds may buffer any negative impacts of noise. This has clinical implications; for example, music interventions in the PICU have been associated with reduced heart rate, improved oxygenation, and decreased behavioural indicators of pain [54]. Patient‐preferred music and familiar voices may introduce a unique child‐centred counterbalance to the otherwise highly medicalised setting.

## 5 Limitations and Implications for Future Research

Data analysis in a scoping review is descriptive only, no formal quality or risk of bias assessment was undertaken; therefore, the strength and certainty of the included evidence could not be evaluated. Although studies from a range of countries were included, more than half were conducted in the United States (*n* = 20, 55.6%), and most originated from high‐income countries. Furthermore, many studies had small sample sizes and may not have adequately accounted for confounders related to illness severity and related treatment which may limit interpretation of relationships between sound and patient outcomes. The multidisciplinary authorship of this review supported interpretation that balanced biomedical and humanistic perspectives; however, key gaps in the evidence remain. Sufficiently powered and reproducible studies are needed to better understand the immediate‐, medium‐ and longer‐term effects of the sound environment on patient, family and staff outcomes. Current research has prioritised measurement of sound levels, with limited attention to the qualitative characteristics of the sound. Analyses that better characterise the sound environment will give a more complete picture of the different types of sound exposure in the PICU. This could include qualitative work exploring experience of the sound.

Future research should also move beyond the assumption that all loud sound is harmful. In addition to reducing noxious sounds, we must investigate how positive auditory inputs influence patient and family wellbeing. A more nuanced understanding of the auditory environment will better inform strategies that both minimise harm and optimise benefit. Clearer evidence linking sound exposure and patient outcomes will enable design and evaluation of targeted interventions in the PICU.

## 6 Conclusion

The PICU is consistently loud and acoustically complex, but the clinical significance of this exposure remains unclear. Evidence linking sound to meaningful patient outcomes is inconsistent and not clearly translated into standard care. Rigorous, patient‐centred research is needed to better understand if modification of the PICU sound environment might improve outcomes.

## Funding

The authors have nothing to report.

## Conflicts of Interest

The authors declare no conflicts of interest.

## Supporting information


**Data S1:** Preferred reporting items for systematic reviews and meta‐analyses extension for scoping reviews (PRISMA‐ScR) checklist.

## Data Availability

Data sharing not applicable to this article as no datasets were generated or analysed during the current study.

## Appendix A Medline (Ovid) Search Strategy

1. noise/ or noise, occupational/
2. (noise or noisy or auditory or sound*).tw,kf,hw
3. 1 or 2
4. Critical Illness/
5. critical care/ or early goal‐directed therapy/
6. Extracorporeal Membrane Oxygenation/
7. (critical* adj3 (care or ill* or injur*)).tw,kf
8. (ECLS or ECMO or extracorporal‐membrane‐oxygen* or extra‐corporal‐membrane‐oxygen* or extracorporal‐oxygen* or extra‐corporal‐oxygen* or extracorporeal‐cardiopulmonary‐resuscitation or extra‐corporeal‐cardiopulmonary‐resuscitation or extracorporeal‐circulation or extra‐corporeal‐Circulation or extracorporeal‐life‐support or extra‐corporeal‐life‐support or extracorporeal‐membraneous‐oxygen* or extra‐corporeal‐membraneous‐oxygen* or extracorporeal‐membrane‐oxygen* or extra‐corporeal‐membrane‐oxygen* or extracorporeal‐membranes‐oxygen* or extra‐corporeal‐membranes‐oxygen* or extracorporeal‐membranooxygenation or extra‐corporeal‐membranooxygenation or extracorporeal‐membranous‐oxygen* or extra‐corporeal‐membranous‐oxygen* or extracorporeal‐membranoxygenation or extra‐corporeal‐membranoxygenation or extracorporeal‐oxygen* or extra‐corporeal‐oxygen* or extracorporeal‐pump‐oxygen* or extra‐corporeal‐pump‐oxygen* or extrapulmonary‐blood‐oxygen* or extra‐pulmonary‐blood‐oxygen* or extrapulmonary‐membrane‐oxygen* or extra‐pulmonary‐membrane‐oxygen* or extrapulmonary‐oxygen* or extra‐pulmonary‐oxygen* or extrapulmonic‐oxygen* or extra‐pulmonic‐oxygen*).tw,kf.
9. exp. Respiration, Artificial/
10. (Artificial‐respiration or Ventilat* or Positive‐pressure‐respiration or Positive‐pressure‐breathing or Continuous‐positive‐airway‐pressure or CPAP or artificial‐respiratory‐support or controlled‐respiration or artificial‐lung*).tw,kf
11. 4 or 5 or 6 or 7 or 8 or 9 or 10
12. (newborn* or new‐born* or baby or babies or neonat* or neo‐nat* or infan* or toddler* or pre‐schooler* or preschooler* or kinder or kinders or kindergarten* or kinder‐aged or boy or boys or girl or girls or child* or paediatric* or paediatric* or school‐age* or schoolage* or schoolchild* or schoolgirl* or schoolboy* or pre‐teen* or preteen* or tween or tweens or pre‐adolescen* or preadolescen* or pre‐puberty or prepuberty or pre‐pubescen* or prepubescen* or adolescen* or youth or youths or teen or teens or teenage* or student*).tw,kf,hw
13. 3 and 11 and 12
14. Intensive Care Units, Paediatric/ or PICU.tw,kf
15. (intensive‐care or ICU or ccu).tw,kf,hw.
16. 14 or (15 and 12)
17. 3 and 16
18. 13 or 17
19. limit 18 to yr. = “2014 ‐Current”

## Appendix B PsycINFO (Ovid) Search Strategy

1. exp auditory stimulation/
2. noise effects/
3. “noise levels (work areas)”/
4. (noise or noisy or auditory or sound*).ti,ab,id,hw.
5. 1 or 2 or 3 or 4
6. critical illness/
7. (critical* adj3 (care or ill* or injur*)).ti,ab,id.
8. (ECLS or ECMO or extracorporal‐membrane‐oxygen* or extra‐corporal‐membrane‐oxygen* or extracorporal‐oxygen* or extra‐corporal‐oxygen* or extracorporeal‐cardiopulmonary‐resuscitation or extra‐corporeal‐cardiopulmonary‐resuscitation or extracorporeal‐circulation or extra‐corporeal‐Circulation or extracorporeal‐life‐support or extra‐corporeal‐life‐support or extracorporeal‐membraneous‐oxygen* or extra‐corporeal‐membraneous‐oxygen* or extracorporeal‐membrane‐oxygen* or extra‐corporeal‐membrane‐oxygen* or extracorporeal‐membranes‐oxygen* or extra‐corporeal‐membranes‐oxygen* or extracorporeal‐membranooxygenation or extra‐corporeal‐membranooxygenation or extracorporeal‐membranous‐oxygen* or extra‐corporeal‐membranous‐oxygen* or extracorporeal‐membranoxygenation or extra‐corporeal‐membranoxygenation or extracorporeal‐oxygen* or extra‐corporeal‐oxygen* or extracorporeal‐pump‐oxygen* or extra‐corporeal‐pump‐oxygen* or extrapulmonary‐blood‐oxygen* or extra‐pulmonary‐blood‐oxygen* or extrapulmonary‐membrane‐oxygen* or extra‐pulmonary‐membrane‐oxygen* or extrapulmonary‐oxygen* or extra‐pulmonary‐oxygen* or extrapulmonic‐oxygen* or extra‐pulmonic‐oxygen*).ti,ab,id.
9. artificial respiration/
10. (Artificial‐respiration or Ventilat* or Positive‐pressure‐respiration or Positive‐pressure‐breathing or Continuous‐positive‐airway‐pressure or CPAP or artificial‐respiratory‐support or controlled‐respiration or artificial‐lung*).ti,ab,id.
11. 6 or 7 or 8 or 9 or 10
12. (newborn* or new‐born* or baby or babies or neonat* or neo‐nat* or infan* or toddler* or pre‐schooler* or preschooler* or kinder or kinders or kindergarten* or kinder‐aged or boy or boys or girl or girls or child* or pediatric* or paediatric* or school‐age* or schoolage* or schoolchild* or schoolgirl* or schoolboy* or pre‐teen* or preteen* or tween or tweens or pre‐adolescen* or preadolescen* or pre‐puberty or prepuberty or pre‐pubescen* or prepubescen* or adolescen* or youth or youths or teen or teens or teenage* or student*).ti,ab,id,hw.
13. 5 and 11 and 12
14. PICU.ti,ab,id.
15. intensive care/
16. (intensive‐care or ICU or CCU).ti,ab,id,hw.
17. 14 or ((15 or 16) and 12)
18. 5 and 17
19. 13 or 18
20. limit 19 to yr=“2014 ‐Current”
